# Medicine

**Published:** 1893-03

**Authors:** J. Michell Clarke


### Progress of the Medical Sciences.

### MEDICINE.

The recent treatment of myxœdema by injections of extract of the thyroid gland opens a new era in therapeutics. No more striking testimony of the value of exact and thorough knowledge of the pathology of a disease, and of the indispensable necessity for the confirmation of pathological observations by experiments on animals, in order to attain to successful treatment, could be afforded. This new discovery in therapeutics is the triumphant outcome of accurate clinical, pathological, and experimental work on the disease in question.

Dr. Ord had established the fact that the constant pathological feature in myxœdema is the atrophy of the thyroid. Dr. Semon, Professors Horsley and Kocher proved that the symptoms of myxœdema could be produced in animals by extirpation of this gland. The experiments of G. Vassale and E. Gley showed that injections of thyroid extract removed the acute symptoms which follow thyroidectomy in dogs. Brown-Séquard and d'Arsonval argued from this that injections of thyroid extract would be beneficial in myxœdema. Dr. Murray was the first to put these data into practical application, and, by successfully carrying out this treatment, to show that a remarkable amelioration could be produced in the symptoms of myxœdema by injections of extract of sheep's thyroid, and that this improvement was maintained so long as the injections were continued. He brought forward his first case in 1891,[2] and in 1892, at the Nottingham meeting of the British Medical

[1] *Op. cit.*, p. 343. [2] *Brit. Med. Journ.*, Oct. 10, 1891.

Association, he[1] was able to report four cases, in all of which most marked improvement had followed the injections. The improvement consisted in loss of the characteristic œdema, recovery of mental and bodily activity, in a rise of temperature, and in loss of weight. Dr. Wallace Beatty,[2] Dr. Ernest Carter,[3] and Dr. Arthur Davies[4] report similar improvement in other cases. The latter observed that when the injections were discontinued for some weeks the patient began to regain his former aspect, and to recover weight, and the old feelings of his myxœdematous state began to return. Dr. G. E. Hale[5] reports four cases, in three of which there was decided improvement, and in one slight, as the result of the injections. He found that three to four weeks' abstention from treatment allowed a certain amount of deterioration to occur. In two cases, in which the blood was examined, no appreciable difference in the red corpuscles was produced. No accurate measurements were made of the amount of urine passed, but three patients stated that there was diuresis. Dr. Murray was unable to trace any definite diuretic effect from the injections in his patients. In a case reported by Dr. E. Mendel[6] the patient felt better, moved more freely, and was more loquacious. The swelling was much diminished. The pulse-rate increased from 60 to 70. The temperature rose from 34.8° C. to 36° C. The amount of urine increased from 1,100 c.c. daily at first to 1,450 c.c., and then to 2,000 c.c. on the fourth day after the injections were begun. A week later the quantity was 1,500 c.c. The amount of urea increased from 14.3 gr. to 20.25 gr. daily. Mr. Hurry Fenwick[7] observed in a case of myxœdema into which he had grafted a sheep's thyroid that the temperature rose to normal (it had been sub-normal previously), and the quantity of urine increased from 20 to 50 ounces. He states that after injections of thyroid juice the urine increases in amount during the next day or the day after, and the effect in the myxœdema case continues for 14 to 21 days. Mr. Fenwick goes on to say: "We are inclined to believe, from work which we have done in this direction, that the theory of the action of the diseased thyroid gland is incorrect, and that the state known as myxœdema depends upon a perverted renal function. We find that the thyroid juice possesses a distinct diuretic action in diseases of the kidney, though apparently it is negative in healthy persons." Mr. Beadles[8] also found that a considerably larger amount of urine was passed, as a consequence of the treatment, by a patient who suffered from advanced myxœdema with delusions and religious melancholia. In this case there was great improvement in the myxœdematous, and as great, or greater, in the mental symptoms. In connection with cases

[1] *Brit. Med. Journ.*, Aug. 27, 1892.
[2] *Ibid.*, Mar. 12, 1892. [3] *Ibid.*, Apr. 16, 1892. [4] *Ibid.*, Aug. 27, 1892.
[5] *Ibid.*, Dec. 31, 1892. [6] *Deutsch. med. Woch.*, Jan. 12, 1893.
[7] *Brit. Med. Journ.*, Oct. 10, 1891.
[8] *Ibid.*, Dec. 24, 1892.

showing pronounced mental change, Drs. Claye Shaw, and Stansfield[1] also report a case of myxœdema with restless melancholia cured by injections, both as to myxœdematous symptoms and mental state. Here there was an increase in the hæmoglobin and in the number of red blood corpuscles. A third instance is given by Dr. Ralph Wickmann,[2] in which symptoms of hypochondriasis and melancholia which accompanied the myxœdematous changes were replaced by cheerfulness, and the myxœdema was relieved.[3]

Two well-marked cases of myxœdema were treated at the Bristol General Hospital under my supervision[4] with injections of thyroid extract. No good result followed; but, in view of the above results, I must now attribute my failure to an error of observation, possibly in the butcher supplying the thymus instead of the thyroid gland.

At a recent meeting of the Bath and Bristol Branch of the British Medical Association, Dr. Watson Williams showed a patient whom he had treated by injections, with complete amelioration of symptoms.

The method of injection was found to be attended in certain cases by some unpleasant symptoms, of which the most important were: (1) inflammation and abscess in the site of the injection, avoided by careful sterilisation of the extract and instruments used; (2) giddiness and headache; (3) faintness, (4) loss of power in the upper extremities. Further experience seems to show that the administration of the thyroid by the mouth is equally effectual, and unattended with the drawbacks and inconveniences of the injection method.

Dr. E. L. Fox, of Plymouth, and Dr. Hector Mackenzie have recorded[5] cases of myxœdema relieved by the administration of thyroid by the mouth. The former gave at first the glycerine extract of thyroid, and afterwards the gland minced and lightly fried. Profuse perspirations and rapid weakness ensued when the patient took a double quantity of the gland by mistake. Dr. Mackenzie administered fresh sheeps' thyroids finely minced. The effects were shown at once in a marked acceleration of the pulse, and a rise of temperature proportional to the quantity of the gland taken and persisting some time after the administration was continued. A suitable dose causes the temperature to rise to the normal, or a little above it. There is a general diminution of the swelling and an amelioration of all the other symptoms accompanying myxœdema. He states[6] that "one gland or half a gland, or the extract therefrom, twice a week is as much as it seems advisable to commence with, and

[1] *Brit. Med. Journ.*, Aug. 27, 1892.
[2] *Deutsch. med. Woch.*, Jan. 12, 1893.
[3] Many of the above were not recent, but old-standing cases, and apparently it is never too late to begin treatment.
[4] See *Brit. Med. Journ.*, Aug. 27, 1892. [5] *Ibid.*, Oct. 29, 1892.
[6] *Lancet*, Jan. 21, 1893.

at a later period the same amount once a week appears to be sufficient." After mincing the gland it is allowed to stand for about half an hour with a few teaspoonsful of water, and then the whole is strained, the juice being squeezed through a piece of muslin, and the expressed fluid added to some beef-tea. The gland may also be given lightly fried, but not thoroughly cooked. Mr. White, the pharmaceutist, of St. Thomas's Hospital, has succeeded in preparing a powder which is tasteless, keeps perfectly, and answers very well so far as it has been tried. These improvements thus constitute a further advance in the method of treatment. Dr. Mackenzie found an acceleration of pulse (from 56 to 116 in one case) and vomiting, which latter may be very troublesome if the remedy is pushed, to be the consequences of an overdose. After his patient first began to get about she suffered from œdema of the feet and legs, which disappeared as the heart grew stronger.

It is very necessary to allow time for the heart to recover from the degenerative changes which often affect its walls in myxœdema, and to insist that the patient when convalescent should for some time lead an inactive life, and very gradually increase the amount of exercise taken, on account of the danger of syncope from failure of the heart's action. In two of Dr. Murray's cases death occurred suddenly from syncope after very great amelioration of the myxœdematous symptoms. One patient died just after walking up a hill more quickly than usual after having recovered from an attack of bronchitis; and the other, in whom signs of degeneration of the cardiac muscle existed, fainted whilst stooping to put on her shoes, and died half an hour afterwards.

At a discussion on myxœdema, at the Edinburgh Medico-Chirurgical Society,[1] several fresh cases of myxœdema were mentioned, in which great amelioration of the symptoms had followed as a result of the thyroid treatment. Dr. Murray stated that if the treatment is stopped the old symptoms are likely to return in all cases.

Dr. Foulis reported a case of his in which death had occurred within twenty-four hours after taking a quarter of a sheep's thyroid. Profuse diarrhœa was set up; the patient became exhausted, comatose, and died so. It was a most pronounced case. This is the only instance in which such an unfortunate result has occurred.

Knowing the close relationship between myxœdema and sporadic cretinism, we should naturally look for good results from the administration of thyroid extract in the latter disease; and at the same meeting Dr. Thomson showed two cases of sporadic cretinism, both of which had been treated by thyroid-feeding with wonderful success.

Dr. W. B. Ransome[2] and Dr. Mackenzie[3] have also tried

[1] *Brit. Med. Journ.*, Feb. 25, 1893. [2] *Ibid.*, Aug. 27, 1892. [3] *Loc. cit.*

injections of thyroid in two cases each of Graves's disease, but without obtaining any beneficial result.

Dr. D. J. Brakenridge[1] has employed the transfusion of human blood in the treatment of pernicious anæmia. He reports five cases, in four of which the effect of the transfusions was good. The other case was too far advanced in the disease to derive any benefit from the single transfusion attempted. From 4 to 6 ounces of blood was the quantity injected, mixed with a 5 per cent. phosphate of soda solution in order to prevent coagulation. The corpuscles in the injected blood would appear not to be disintegrated after injection, for when the transfusion was satisfactorily performed free from all complications it was found that there was an increase in the number of corpuscles per c. mm. in the patient's blood closely proportionate to the number of corpuscles injected. "The red corpuscles in the blood of a healthy donor are really added to the blood of the recipient without any perceptible destruction of them in the process." The details of the operation require to be most carefully carried out, as mistakes in the preparation of the solution, instruments, and vessels employed, or too great rapidity in carrying out the injection, may be the causes of alarming symptoms. We refer our readers to the original paper for full information on these points. Dr. Brakenridge says that in none of his cases, even when a distinct mistake has occurred, has any accident leaving a permanent effect happened; nor has any other than a good result ultimately followed the transfusion in any of his nine transfusions. He concludes:

(1) If all the necessary precautions are strictly adhered to, the operation is perfectly safe.

(2) Quite healthy blood with living blood corpuscles can be added to the diseased blood of the patient.

(3) This blood exerts a beneficial influence both on the blood with which it is mixed and on the blood-forming organs, as shown by the fact that sooner or later the blood corpuscles begin to increase in excess of those added by the transfusions, with a corresponding increase in the amount of hæmoglobin, and a disappearance of the abnormalities in the forms and varieties of the corpuscles.

The author thinks that these facts are opposed to the view that an abnormally destructive activity of a disordered liver is the only or the main pathological condition in pernicious anæmia, and favours the conclusion that—

(1) The real condition of the blood in this disease is a delicacy and tendency to early death of the red corpuscles.

[1] *Edin. Med. Journ.*, Nov., 1892.

(2) The probable starting point of this condition is some functional weakness of the blood-forming organs, which may be due to various possible causes.

(3) The abnormalities in the shape and size of the corpuscles point to some such imperfect genesis.

(4) Consequently, without any abnormally increased destructive force in the portal system and organs—it being a normal function of the liver-cells to destroy the red corpuscles—a greatly increased death-rate of these delicate and short-lived corpuscles takes place.

The introduction by transfusion of a considerable amount of healthy blood acts beneficially in the way pointed out above.

Dr. Sturges[1] states, relying on 100 cases of heart disease examined *post mortem* at the Great Ormond Street Children's Hospital, that heart affections in childhood may be divided into two great divisions: one, peri-endocarditis, which is rheumatic, and sufficiently described as rheumatic carditis; the other, pericarditis, due to a variety of causes, and in addition, rarely, recent endocarditis. A further proviso must be added that rheumatism accounts for more than half in the total of children's heart disease. Heart disease in the strictest sense is almost wholly rheumatic in them, and almost confined to non-tuberculous children. What remains applies to the heart's investment, and is an accompaniment of morbid conditions wherein the pericardium sympathises, not with the endocardium, but with the neighbouring serous membrane of the lung. The non-rheumatic heart affections of children are insidious in character, and mostly consecutive to other diseases, whose natural course they hardly modify; thus, of 46 patients, 11 had empyema, 10 tubercle, 4 diphtheria, and 3 pneumonia. The rest were associated with meningitis, nephritis, and septicæmia. Frequent recurrences of rheumatism are almost certain to injure the heart, and in childhood recurrence is common; the younger the subject the greater the liability to recurrence and the more closely are the attacks crowded together. Out of 56 cases, in only 11 did fatal heart disease result from a single rheumatic attack, so far as the history could be trusted; nor was this absolutely certain in more than four or five of the number. It is in the later attacks of rheumatic arthritis that physical signs in respect of the heart are most trustworthy; the chief contrast between the child and the man being, that in the former pericardial signs are far more frequent, whilst endocardial signs remain for a somewhat longer space of time of doubtful significance. In forming a judgment the real difficulty is less in diagnosis than in prognosis. In rheumatism, peri- and endo-carditis go nearly always together, so that the presence of

[1] *Lancet*, 1892, vol. i., p. 621.

the former entails the latter, whilst pericardial inflammation is shown by pericardial sound. Dr. Sturges finds that not only a "to-and-fro" rub, but also creaking, scraping, or various other exocardial sounds, may occur with extensive pericardial adhesions. In the case of children who die after repeated rheumatic carditis, with or without a rub, it is the rule to find adhesions, and at the same time it is the rule to hear an exocardial rub. Very extensive adhesion will often permit very obvious exocardial sound, and examples occur where this is absent whilst the pericardium is rough and lymph-covered. If we add, as is generally admitted, that friction sound may be present where there is no lymph and no roughness, then the teaching of the text books, that the successive stages of pericarditis may be followed by the ear, is erroneous.

J. MICHELL CLARKE.

